# Step length and fall risk in adults with chronic kidney disease: a pilot study

**DOI:** 10.1186/s12882-022-02706-w

**Published:** 2022-02-22

**Authors:** Atsumi Kimura, William Paredes, Rima Pai, Hina Farooq, Rupinder S. Buttar, Matthew Custodio, Samhitha Munugoti, Sonia Kotwani, Lovepreet S. Randhawa, Solomon Dalezman, Antonio C. Elters, Kate Nam, Jose S. Ibarra, Sandheep Venkataraman, Matthew K. Abramowitz

**Affiliations:** 1 Department of Medicine, Albert Einstein College of Medicine, 1300 Morris Park Avenue, Ullmann 615, Bronx, NY USA; 2 Institute for Aging Research, Albert Einstein College of Medicine, Bronx, NY USA; 3 Diabetes Research Center, Albert Einstein College of Medicine, Bronx, NY USA; 4 Fleischer Institute for Diabetes and Metabolism, Albert Einstein College of Medicine, Bronx, NY 10461 USA

**Keywords:** Chronic kidney disease, Dialysis, Gait, Gait speed, Step length, Fall, Fall risk

## Abstract

**Background:**

Patients with chronic kidney disease commonly experience gait abnormalities, which predispose to falls and fall-related injuries. An unmet need is the development of improved methods for detecting patients at high risk of these complications, using tools that are feasible to implement in nephrology practice. Our prior work suggested step length could be such a marker. Here we explored the use of step length as a marker of gait impairment and fall risk in adults with chronic kidney disease.

**Methods:**

We performed gait assessments in 2 prospective studies of 82 patients with stage 4 and 5 chronic kidney disease (*n* = 33) or end-stage renal disease (ESRD) (*n* = 49). Gait speed and step length were evaluated during the 4-m walk component of the Short Physical Performance Battery (SPPB). Falls within 6 months prior to or following enrollment were identified by questionnaire. Associations of low step length (≤47.2 cm) and slow gait speed (≤0.8 m/s) with falls were examined using logistic regression models adjusted for demographics and diabetes and peripheral vascular disease status.

**Results:**

Assessments of step length were highly reproducible (*r *= 0.88, *p* < 0.001 for duplicate measurements at the same visit; *r* = 0.78, *p* < 0.001 between baseline and 3-month evaluations). Patients with low step length had poorer physical function, including lower SPPB scores, slower gait speed, and lower handgrip strength. Although step length and gait speed were highly correlated (*r* = 0.73, *p* < 0.001), one-third (*n* = 14/43) of patients with low step length did not have slow gait speed. Low step length and slow gait speed were each independently associated with the likelihood of falls (odds ratio (OR) 3.90 (95% confidence interval (CI) 1.05–14.60) and OR 4.25 (95% CI 1.24–14.58), respectively). Compared with patients who exhibited neither deficit, those with both had a 6.55 (95% CI 1.40–30.71) times higher likelihood of falls, and the number of deficits was associated with a graded association with falls (p trend = 0.02). Effect estimates were similar after further adjustment for ESRD status.

**Conclusions:**

Step length and gait speed may contribute additively to the assessment of fall risk in a general adult nephrology population.

## Background

Decreased physical function, falls, and fall-related injuries are common among adults with chronic kidney disease (CKD) [1–3]. Fall injuries are a leading cause of hospitalization and death in this population and are associated with a considerably lower likelihood of receiving a kidney transplant [4–7]. Detection of patients at high risk of these complications remains suboptimal; it is imperative to develop methods that are feasible to implement in clinical nephrology practice. One promising approach involves the detection of gait abnormalities [8]. Gait speed, for example, is simple to measure, takes less than 1 min to complete, and is associated with both functional and cognitive outcomes [3, 9]. In addition, our work and that of others have identified other abnormalities of gait among adults with CKD [10, 11]; these are present even among individuals with preserved gait speed and are associated with elevated risk of falls and development of cognitive impairment, the latter of which can further exacerbate fall risk [10, 12, 13].

Certain aspects of gait dysfunction in CKD are measurable using advanced technology and quantitative methods; others are detectable on clinical examination. For example, shortened steps and loss of balance manifested as swaying while walking are manifestations of CKD-related gait dysfunction that can be detected on gait examination and are associated with a higher risk of falling, independent of other fall risk factors [10, 12]. Although many clinicians may lack familiarity with the gait examination, step length can be measured quantitatively, and thus has the potential for easier implementation in clinical practice. Therefore, we sought to examine the utility of a step length measurement performed simultaneously with gait speed as an additional gait metric in CKD, with falls as the primary outcome of interest. We hypothesized that shorter step length would be associated with a higher likelihood of falling, and that it would provide additive information when combined with gait speed. Because gait is not assessed currently as part of nephrology care, and gait parameters other than speed have not been measured in studies of patients with advanced CKD, we pooled data from 2 studies in which we have conducted such assessments.

## Methods

### Study population

**Fig. 1:**
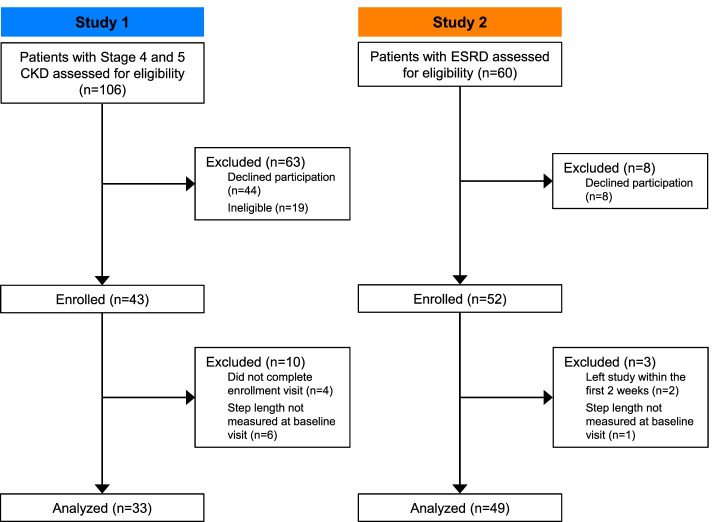
Flow diagram of study participation. CKD, chronic kidney disease; ESRD, end-stage renal disease

We combined data from two prospective observational studies: a study of physical function in patients with stage 4 and 5 CKD (Study 1), and a physical activity monitoring study in patients with end-stage renal disease (ESRD) receiving hemodialysis (Study 2) (Fig. 1). The study protocols were approved by the Institutional Review Board of the Albert Einstein College of Medicine and conducted in accordance with the Declaration of Helsinki. Before inclusion in each study, written informed consent was provided by all participants. Study 1 enrolled ambulatory patients ≥21 years of age with an estimated glomerular filtration rate (eGFR) < 30 mL/min/1.73m^2^ who were not receiving renal replacement therapy. Exclusion criteria included bilateral lower extremity amputations, use of immunosuppressive medications in the prior 3 months, an active cancer diagnosis, or receiving treatment for cancer. Study 2 enrolled ambulatory patients ≥18 years of age who had been receiving thrice-weekly hemodialysis for at least 3 months and were willing to wear a wrist-worn accelerometer for the duration of the study. Visits occurred every 3 months in Study 1 and monthly in Study 2.

### Physical function, gait, and falls assessment

Physical function was assessed at baseline in both studies using the Short Physical Performance Battery (SPPB) [14] and handgrip strength, which was measured using a Jamar dynamometer. In Study 2, assessments occurred in the dialysis unit following hemodialysis treatments. As part of the SPPB, participants completed a 4-m walk twice at usual pace. The 4-m walking course was defined by two thin pieces of tape on the floor visible only to the tester placed 4 m apart. The subject’s starting point was defined by two traffic cones placed 1 m before the start tape and 1 m after the end tape. This was done to eliminate walking acceleration and deceleration effects. The subject was asked to walk from the start cone to the end cone at their normal pace. An observer stood at an optimal viewing vantage off to the side between the cones. The observer started the stopwatch whenever the subject’s foot landed on or went over the start marker. The very next step (i.e. when the opposite foot touched the ground) was counted as step #1, the subsequent step was counted as step #2, and so forth. The counting continued until either foot touched or crossed over the end tape, at which point the stopwatch was concurrently toggled off. The walking trial was then repeated in the reverse direction. We did not conduct assessments on participants with recent injuries that could affect their performance.

The walk completed in the shortest time was used for measurements of gait speed and step length. The time required to complete the walk was used to calculate gait speed. The number of steps taken during the walk was used to compute step length, which was calculated as 4 m divided by number of steps. Slow gait speed was defined as ≤0.8 m/s [15]. Low step length was defined as step length equal to or below the median in this study sample. For the sit-to-stand component of the SPPB, participants who were unable to complete 5 repetitions were assigned a time of 60 s for the purpose of the current analysis. At the baseline visit in each study, participants were asked the question “Have you had any falls in the past 6 months?” Falls were then assessed prospectively every 3 months in Study 1 and monthly in Study 2. For participants who reported falling, the number of falls was also recorded. Because Study 2 was 6 months in duration, only data collected through the 6-month follow-up visit in Study 1 were included in this analysis. Participants were categorized as having experienced a fall if they reported falling during the 6 months prior to the baseline visit or during follow-up.

### Statistical analysis

Baseline characteristics were compared between participants with and without low step length using χ^2^ or Fisher’s exact tests for categorical variables and two-tailed t-tests for continuous variables. Correlations of gait and physical function metrics were examined using Pearson correlation coefficients. Associations with likelihood of falls were examined using logistic regression models adjusted for age, sex, race/ethnicity, and diabetes mellitus, peripheral vascular disease, and ESRD status. To examine the associations of gait parameters with fall incidence prospectively, marginal generalized estimating equations-based negative binomial models were fit to account for with-person correlation.

## Results

### Participant characteristics and gait markers

**Fig. 2:**
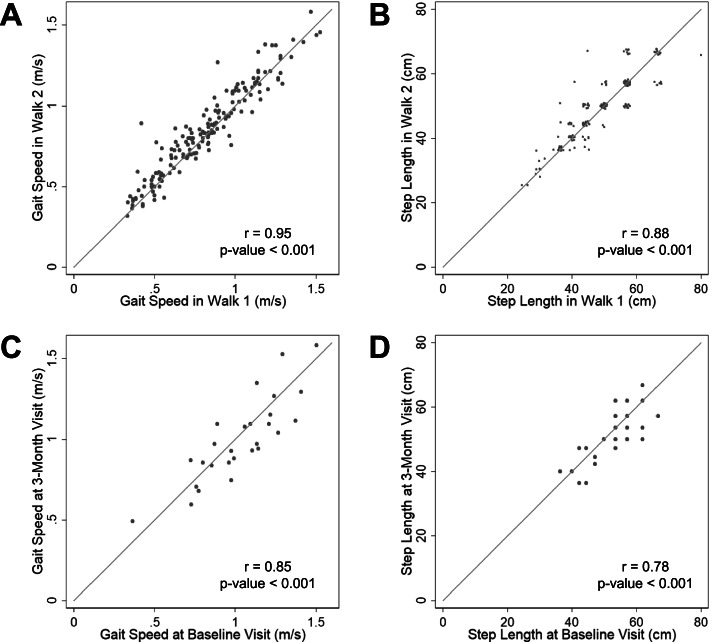
Reproducibility of step length measurements. **A**,**B** Correlations of gait speed (**A**) and step length (**B**) in walks 1 and 2 of 4-m walk assessment. Due to overlapping values in B, a small jitter (random noise) was applied to enable visualization of all data points. **C**,**D** Correlations of gait speed (**C**) and step length (**D**) at baseline visit and 3-month visit in patients with non-dialysis dependent CKD

**Fig. 3:**
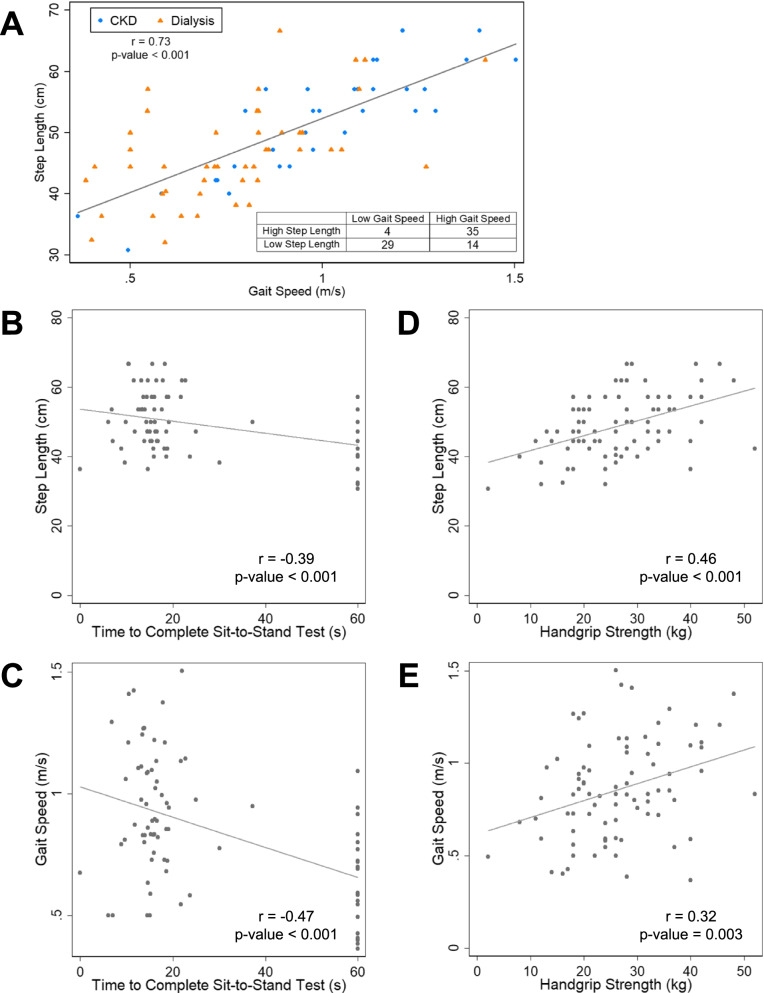
Associations between step length, gait speed and physical function measures. **A** Correlation of step length with gait speed, and cross-classification of participants by low step length and slow gait speed status. **B**,**C** Correlations of step length (**B**) and gait speed (**C**) with the time to complete the 5-repetition sit-to-stand test. **D**,**E** Correlations of step length (**D**) and gait speed (**E**) with handgrip strength

The study sample included 82 CKD patients (43 men and 39 women), 49 of whom were receiving dialysis (Table 1). The mean age was 60.3 ± 13.1 years, with a range from 25 to 86; 23% (*n* = 19/82) were aged < 50 years. The median gait speed was 0.83 m/s and median step length was 47.2 cm. Patients with ESRD had poorer physical function than patients with non-dialysis dependent CKD (SPPB score 7.4 ± 2.6 vs. 8.2 ± 2.6, *p* = 0.1; gait speed 0.8 ± 0.2 vs. 1.0 ± 0.3 m/s, *p* < 0.001; handgrip strength 22.2 ± 12.5 vs. 28.2 ± 10.2 kg, *p* = 0.03), although they were not older (age 59.0 ± 13.1 vs. 62.3 ± 13.1 years old, *p* = 0.3). For both gait speed and step length, we noted strong within-visit correlations between measurements taken during the two 4-m walks conducted at each visit (Fig. 2A, B). To further examine reproducibility, in Study 1 gait assessment was repeated 3 months following the baseline evaluation, and we also noted strong correlations between these timepoints (Fig. 2C, D). Compared with patients with high step length, those with low step length were more likely to have diabetes mellitus, peripheral vascular disease, and ESRD (Table 1). They had poorer physical function, with lower scores on the SPPB, slower gait speed, and lower handgrip strength. Body mass index and height did not differ based on step length. Among non-dialysis dependent patients, the mean eGFR was 21.7 ± 8.2 mL/min/1.73m^2^; eGFR also did not differ between the two groups. There was a strong positive correlation between step length and gait speed (Fig. 3A). Similarly, both lower step length and slower gait speed correlated with longer time to complete the 5-repetition sit-to-stand test (Fig. 3B, C) and with weaker handgrip strength (Fig. 3D, E). Although nearly all patients with slow gait speed also had low step length (*n* = 29/33), 33% (*n* = 14/43) of patients with low step length did not have slow gait speed. Among the subgroup of patients who did not meet the definition for slow gait speed, those with low step length had slower gait speed (Table 2) than patients with high step length. Among this subgroup, other physical function measures did not differ based on step length, although SPPB scores and handgrip strength were numerically lower among those with low step length (Table 2).

**Table 1:** Baseline characteristics by step length

	Low Step Length (*n* = 43)	High Step Length (*n* = 39)	*p*-value
Age (years)	61.5 ± 12.7	59.0 ± 13.6	0.39
Women – n (%)	23 (53.5)	16 (41.0)	0.26
Race – n (%)			
Black	24 (55.8)	21 (53.9)	
Hispanic	5 (11.6)	11 (28.2)	0.10
Other	14 (32.6)	7 (18.0)	
Body Mass Index (kg/m^2^) (*n* = 81)	29.8 ± 6.5	31.1 ± 7.8	0.44
Height (cm)	167.1 ± 1.4	169.5 ± 8.9	0.23
Diabetes – n (%)	27 (62.8)	17 (43.6)	0.08
Hypertension – n (%)	40 (93.0)	37 (94.9)	0.73
Peripheral Vascular Disease – n (%)	13 (30.2)	4 (10.3)	0.03
ESRD – n (%)	32 (74.4)	17 (43.6)	0.004
eGFR (mL/min/1.73 m^2^) ^a^	22.3 ± 8.1	21.4 ± 8.5	0.78
SPPB Score (categorical) – n (%)			
0–6	17 (39.5)	4 (10.3)	0.002
7–9	20 (46.5)	19 (48.7)	
10–12	6 (14.0)	16 (41.0)	
SPPB Score (continuous)	6.6 ± 2.8	8.9 ± 1.8	< 0.001
Gait Speed (m/s)	0.7 ± 0.2	1.0 ± 0.2	< 0.001
Sit-to-Stand Time (s)	33.1 ± 22.4	21.5 ± 15.8	0.009
Handgrip Strength (kg)	23.0 ± 9.4	30.1 ± 8.2	< 0.001

*Abbreviations*: *ESRD* End Stage Renal Disease, *eGFR* Estimated Glomerular Filtration Rate, *SPPB* Short Physical Performance Battery

Low step length defined as step length ≤ 47.2 cm

^a^ eGFR values only available for participants who were not receiving dialysis (*n* = 33; low step length = 11, high step length = 22)

**Table 2:** Physical Function Measures among Patients with Normal Gait Speed

	Low Step Length (*n* = 14)	High Step Length (*n* = 35)	*p*-value
SPPB Score (categorical) – n (%)			
0–6	0 (0)	2 (5.7)	0.3
7–9	10 (71.4)	17 (48.6)	
10–12	4 (28.6)	16 (45.7)	
SPPB Score (continuous)	8.8 ± 1.2	9.2 ± 1.7	0.4
Gait Speed (m/s)	0.9 ± 0.1	1.1 ± 0.2	0.009
Handgrip Strength (kg)	25.0 ± 10.9	30.1 ± 8.5	0.09
Time to Complete Sit-To-Stand Test (s)	22.1 ± 16.4	19.7 ± 13.5	0.6

*Abbreviations*: *SPPB* Short Physical Performance Battery

Low step length defined as step length ≤ 47.2 cm

### Associations of gait markers with falls

We next examined associations with a history of falling. The median follow-up time to the final assessment included in our analyses was 189 days (interquartile range, 176–197). Participants who reported at least one fall had significantly lower step length than those without falls (43.8 ± 7.3 vs. 50.7 ± 8.8 cm, *p* = 0.0016). Those with low step length were 6 times more likely (OR 6.30; 95% CI, 1.90–20.89) to have reported a fall than participants with high step length (Table 3). After adjustment for demographic characteristics and presence of diabetes and peripheral vascular disease, participants with low step length had 3.90 (95% CI, 1.05 to 14.60) higher odds of reporting a fall compared to participants with high step length. This was further attenuated by adjustment for ESRD status (OR 3.18, 95% CI, 0.81 to 12.43). Similar findings were seen with gait speed: after multivariable adjustment including diabetes and peripheral vascular disease status, patients with slow gait speed were 4.25 (95% CI, 1.24 to 14.58) times more likely to experience a fall; this was attenuated by further adjustment for ESRD status (OR 3.48, 95% CI, 0.94–12.81).

**Table 3:** Likelihood of Experiencing a Fall by Step Length and Gait Speed

	# Reporting Falls – n (%)	Unadjusted	Model 1	Model 2	Model 3
		OR (95% CI)	OR (95% CI)	OR (95% CI)	OR (95% CI)
Step Length					
High (*n* = 39)	4 (10.3)	Ref	Ref	Ref	Ref
Low (*n* = 43)	18 (41.9)	6.30 (1.90–20.89)	5.65 (1.65–19.36)	3.90 (1.05–14.60)	3.18 (0.81–12.43)
Gait Speed					
High (*n* = 49)	7 (14.3)	Ref	Ref	Ref	Ref
Low (*n* = 33)	15 (45.5)	5.00 (1.74–14.34)	5.05 (1.64–15.53)	4.25 (1.24–14.58)	3.48 (0.94–12.81)
Combined Step Length and Gait Speed					
Both High (*n* = 35)	3 (8.6)	Ref	Ref	Ref	Ref
One Low / One High (*n* = 18)	5 (27.8)	4.10 (0.85–19.72)	4.49 (0.89–22.71)	3.73 (0.66–21.03)	3.18 (0.55–18.55)
Both Low (*n* = 29)	14 (48.3)	9.96 (2.48–39.95)	9.02 (2.16–37.65)	6.55 (1.40–30.71)	5.30 (1.05–26.82)
P for trend		0.001	0.002	0.02	0.04
Gait Speed with Addition of Step Length					
High Gait Speed, High Step Length (*n* = 35)	3 (8.6)	Ref	Ref	Ref	Ref
High Gait Speed, Low Step Length (*n* = 14)	4 (28.6)	4.27 (0.81–22.37)	4.19 (0.75–23.27)	3.12 (0.49–19.82)	2.78 (0.43–17.94)
Low Gait Speed, High or Low Step Length (*n* = 33) ^a^	15 (45.5)	8.89 (2.26–34.89)	8.77 (2.12–36.26)	6.75 (1.45–31.35)	5.50 (1.09–27.87)
P for trend		0.002	0.003	0.02	0.04

Low step length defined as step length ≤ 47.2 cm. Low gait speed defined as ≤0.8 m/s

Model 1 adjusted for age, sex, and race

Model 2 adjusted for age, sex, race, diabetes mellitus, and peripheral vascular disease

Model 3 adjusted for age, sex, race, diabetes mellitus, peripheral vascular disease, and end stage renal disease

^a^ Low gait speed was not stratified by step length due to the small number of participants with high step length

We considered whether low step length and slow gait speed provided additive information (Table 3). Compared with participants who met neither criteria, those who met both were 10 times more likely to have reported a fall (OR 9.96; 95% CI, 2.48–39.95). This remained significant after multivariable adjustment for demographic characteristics and presence of diabetes and peripheral vascular disease (OR 6.55; 95% CI, 1.40 to 30.71) as well as additional adjustment for ESRD status (OR 5.30; 95% CI, 1.05 to 26.82), and there was a graded association based on the number of criteria met. Lastly, we investigated the effect of adding step length to the information captured by gait speed. Among patients with normal gait speed, those with low step length had a 4.27 (95% CI, 0.81 to 22.37) higher odds of reporting a fall during the study period; compared with patients with normal gait speed and high step length, those with low gait speed had 8.89 (95% CI, 2.26 to 34.89) times higher likelihood of falling. After full multivariable adjustment including ESRD status, there remained a trend of graded fall risk when classifying patients based on gait speed and step length status (*p* = 0.04). Of note, we were unable to further stratify patients with low gait speed based on step length because nearly all such individuals had low step length.

Finally, we prospectively examined associations of these gait markers with fall incidence by restricting the analysis to falls reported after the baseline assessment. Overall, 11 participants experienced 32 falls during follow-up. After adjustment for demographic characteristics, both low step length and low gait speed were associated with higher fall incidence (Table 4). Following further adjustment for diabetes and peripheral vascular disease status, there remained a significant graded increase in fall risk in association with greater number of gait abnormalities, whether based on the number of abnormalities or by adding low step length status to gait speed status (Table 4). Associations were no longer significant after additional adjustment for ESRD status, but point estimates were similar to those observed in the primary analyses in Table 3.

**Table 4:** Risk of Future Falls by Step Length and Gait Speed

	# Experiencing a Fall / # of Falls	Falls per 10-person months – mean (SD)	Unadjusted	Model 1	Model 2	Model 3
			IRR (95% CI)	IRR (95% CI)	IRR (95% CI)	IRR (95% CI)
Step Length						
High (*n* = 37)	2 / 5	0.2 (0.9)	Ref	Ref	Ref	Ref
Low (*n* = 39)	9 / 27	1.2 (3.1)	6.00 (1.38–26.04)	5.88 (1.35–25.58)	3.48 (0.84–13.34)	2.37 (0.55–10.13)
Gait Speed						
High (*n* = 46)	3 / 9	0.3 (1.2)	Ref	Ref	Ref	Ref
Low (*n* = 30)	8 / 23	1.3 (3.3)	7.96 (2.18–29.13)	7.04 (1.92–25.88)	4.72 (1.34–16.59)	2.84 (0.70–11.47)
Combined Step Length and Gait Speed						
Both High (*n* = 33)	1 / 3	0.1 (0.8)	Ref	Ref	Ref	Ref
One Low / One High (*n* = 17)	3 / 8	0.8 (1.8)	7.77 (0.71–85.60)	7.45 (0.70–78.71)	5.37 (0.54–53.00)	3.65 (0.37–36.11)
Both Low (*n* = 26)	7 / 21	1.4 (3.5)	18.85 (2.06–172.10)	17.18 (1.94–151.87)	10.19 (1.17–88.66)	5.74 (0.60–54.80)
P for trend			0.009	0.01	0.04	0.13
Gait Speed with Addition of Step Length						
High Gait Speed, High Step Length (*n* = 33)	1 / 3	0.1 (0.8)	Ref	Ref	Ref	Ref
High Gait Speed, Low Step Length (*n* = 13)	2 / 6	0.7 (1.9)	5.91 (0.45–78.28)	5.92 (0.46–76.41)	3.90 (0.33–46.17)	3.14 (0.28–35.64)
Low Gait Speed, High or Low Step Length (*n* = 30) ^a^	8 / 23	1.3 (3.3)	18.20 (2.00–165.87)	16.23 (1.81–145.52)	10.19 (1.18–87.90)	5.80 (0.60–55.62)
P for trend			0.01	0.01	0.04	0.13

*Abbreviations*: *IRR* Incidence Rate Ratio

Low step length defined as step length ≤ 47.2 cm. Low gait speed defined as ≤0.8 m/s

Model 1 adjusted for age, sex, and race

Model 2 adjusted for age, sex, race, diabetes mellitus, and peripheral vascular disease

Model 3 adjusted for age, sex, race, diabetes mellitus, peripheral vascular disease, and end stage renal disease

^a^ Low gait speed was not stratified by step length due to the small number of participants with high step length

## Discussion

In our analyses of 82 adults with CKD, we found that step length was associated with established measures of physical function and was lower among dialysis-dependent patients. Both slow gait speed and low step length were associated with higher likelihoods of experiencing a fall, and fall risk was further increased when both characteristics were present. Among patients with normal gait speed, the odds of experiencing a fall were numerically higher in those with low step length compared to high step length, indicating that low step length itself may point to higher fall risk. In addition, the effect estimates for slow gait speed were of greater magnitude when the comparison was with participants with normal gait speed and high step length, as opposed to normal gait speed alone. This could reflect improved classification of fall risk by incorporating information on step length. The point estimates remained elevated even after adjusting for ESRD status, adding to the potential utility of step length in predicting fall risk.

Overall, our findings suggest that step length could be a promising marker of gait abnormalities and fall risk in CKD patients independent of clinical characteristics such as age, diabetes, and ESRD. The use of step length could provide additional value in capturing patients with possible gait impairments who exhibit normal gait speed, which was seen in one-third of our patients with low step length. Our data suggest that these patients may have subtle impairment in physical function that is not captured by the slow gait speed definition. This is an important avenue for future investigation. While there is some evidence that spatiotemporal gait markers are more sensitive parameters to assess fall risk, the feasibility of incorporating such methods in routine clinical care is unclear due to financial and resource constraints [8]. Alternatively, step length, combined with gait speed, could serve as a simple, inexpensive, yet informative gait metric in nephrology practice. The high correlations between step length measurements taken at the same visit and across subsequent visits substantiate the reliability of the marker, and the reproducibility of both step length and gait speed shows that an examiner can measure them simultaneously without loss of precision.

This work builds on our prior description of gait abnormalities in older adults with CKD [10, 12]. In that cohort, lower eGFR was associated with slower gait speed, shorter stride length, and abnormalities of the gait cycle suggestive of impaired balance. A clinically detectable gait phenotype based partly on the presence of short steps was associated with the risk of falls as well as fall-related injuries [10], and with cognitive impairment [12], which is itself a risk factor for falls [13]. Thus, in both this study and our prior work, more advanced kidney disease was associated with greater gait dysfunction, which in turn was associated with a higher risk of falls. Although our focus here is the identification of fall risk, it is also worth noting that these abnormalities of gait may mediate the occurrence of falls in patients with CKD and could partly explain the further elevated risk of falls among patients with ESRD [2].

Several mechanisms could account for shortened step length in CKD and its association with falls. To the extent that step length is highly correlated with gait speed, low step length may be due to the same processes that have been hypothesized to contribute to slow walking speed in the setting of aging [16]. An increased attentional component of gait could unmask other CKD-associated deficits such as neuropathy and balance impairment [17, 18], manifesting in shortened steps. This possibility is supported by enhanced cognitive-motor interference in older adults with CKD [19, 20]. Effects of cerebrovascular disease and other alterations of brain function associated with CKD could also negatively impact the gait cycle; indeed, the previously described gait phenotype was associated with brain atrophy in regions linked with both motoric and cognitive control of gait [12]. Shortened steps could also reflect adjustment to the energy cost of walking in patients with reduced exercise tolerance and impaired muscle bioenergetics [21–23]. The correlations of step length with sit-to-stand time and handgrip strength suggest an important contribution of muscle force generation to step length in patients with advanced CKD. Impaired muscle function and weakness could impact self-selected step length through effects on contraction velocity, balance, joint instability, or the metabolic cost of walking [22–25]. That low step length seems associated with both slowness of gait and weakness potentially links this gait abnormality with frailty status, which is highly prevalent in patients with advanced non-dialysis CKD and in those receiving dialysis [18, 26]. Therefore, step length, like gait speed, may be a gait metric that integrates the functional impacts of a number of neurologic, cardiovascular, and muscular sequelae of CKD.

Several limitations of this pilot study should be noted. First, we defined low step length based on the median obtained from our sample. Future investigation is needed to determine whether this is the optimal method to stratify patients based on step length. In addition, due the brevity of the walk (i.e. 4 m), there was a limited number of combinations of step counts and step lengths as evidenced by overlapping data points seen in Fig. 2B. Moreover, given the modest sample size in this initial investigation, the outcome measure included both retrospective and prospective assessment of falls. It is worth noting that self-report of falls in the previous 6 months is a validated and widely used assessment of fall risk [27]. Nevertheless, this approach could introduce bias related to the ascertainment of falls. To address this possibility, we performed analyses limited to prospectively determined falls; these produced similar effect estimates, lessening concern about the primary analytic results. There was also a difference in recall period for fall history during prospective assessments in Studies 1 and 2, where patients in the former study recalled falls in the past 3 months and patients in the latter recalled falls in the past month. Therefore, the potential impact of recall bias may have differed based on the study in which the patient was enrolled. There was also a lack of information on other fall risk factors such as cognition; we were unable to adjust for these in our analyses. Another important limitation is that the investigators conducting the SPPB were not blinded to subjects’ fall status. However, the protocol was standardized and thus unlikely to have been impacted by this knowledge. Among ESRD patients, physical function testing occurred after hemodialysis treatments; post-dialysis fatigue could have impacted performance and could account for some of the differences between CKD and ESRD patients. Finally, manual assessment of average step length is less precise than automated instrumental methods, and its accuracy relative to a gold standard measurement remains to be determined. These concerns are ameliorated somewhat by the excellent reproducibility we observed. In addition, this manual method does not capture gait variability parameters which could yield additional prognostic information. Future prospective studies are needed to address such questions.

## Conclusion

In conclusion, step length could provide clinically useful information regarding gait abnormalities and fall risk that may not be elucidated with gait speed alone. To our knowledge, this is the first report to explore the use of step length as a gait parameter in a general CKD population. Additionally, in contrast to our previous work that focused on older adults (i.e. ≥65 years) who had less advanced CKD and were not receiving hemodialysis, the present study included a younger cohort and both dialysis- and non-dialysis dependent CKD patients. Further investigation is warranted to assess the prognostic value of step length in the context of falls and fall-related injuries in CKD patients.

## Data Availability

The datasets generated and/or analyzed during the current study are available from the corresponding author on reasonable request.

## Abbreviations

CKD: Chronic kidney disease
ESRD: End-stage renal disease
eGFR: Estimated glomerular filtration rate
SPPB: Short physical performance battery
